# Consultant Input in Acute Medical Admissions and Patient Outcomes in Hospitals in England: A Multivariate Analysis

**DOI:** 10.1371/journal.pone.0061476

**Published:** 2013-04-17

**Authors:** Derek Bell, Adrian Lambourne, Frances Percival, Anthony A. Laverty, David K. Ward

**Affiliations:** 1 Imperial College, London, United Kingdom; 2 pH Associates Ltd, Marlow, United Kingdom; 3 Department of Primary Care and Public Health, Imperial College, London, United Kingdom; 4 South London Healthcare NHS Trust, Queen Elizabeth Hospital, Woolwich, United Kingdom; Universidad Peruana Cayetano Heredia, Peru

## Abstract

Recent recommendations for physicians in the UK outline key aspects of care that should improve patient outcomes and experience in acute hospital care. Included in these recommendations are Consultant patterns of work to improve timeliness of clinical review and improve continuity of care. This study used a contemporaneous validated survey compared with clinical outcomes derived from Hospital Episode Statistics, between April 2009 and March 2010 from 91 acute hospital sites in England to evaluate systems of consultant cover for acute medical admissions. Clinical outcomes studied included adjusted case fatality rates (aCFR), including the ratio of weekend to weekday mortality, length of stay and readmission rates. Hospitals that had an admitting Consultant presence within the Acute Medicine Unit (AMU, or equivalent) for a minimum of 4 hours per day (65% of study group) had a lower aCFR compared with hospitals that had Consultant presence for less than 4 hours per day (p<0.01) and also had a lower 28 day re-admission rate (p<0.01). An ‘all inclusive’ pattern of Consultant working, incorporating all the guideline recommendations and which included the minimum Consultant presence of 4 hours per day (29%) was associated with reduced excess weekend mortality (p<0.05). Hospitals with >40 acute medical admissions per day had a lower aCFR compared to hospitals with fewer than 40 admissions per day (p<0.03) and had a lower 7 day re-admission rate (p<0.02). This study is the first large study to explore the potential relationships between systems of providing acute medical care and clinical outcomes. The results show an association between well-designed systems of Consultant working practices, which promote increased patient contact, and improved patient outcomes in the acute hospital setting.

## Introduction

Acute medicine in the UK, Europe and Australia has developed over the last 15 years in response to the increasing number of medical admissions, concerns over the quality of acute care and other external pressures, including the European Working Time Directive. In 2007, the comprehensive Acute Medicine Task Force report [1] made several recommendations for the provision of high quality systems for patient care in the acute setting. We have previously published an audit of compliance with these standards in acute hospitals [2] and demonstrated that many of the recommendations had at best only been partially implemented. While there is some evidence to support these best practice recommendations [3] there has been minimal evaluation of the impact of different Consultant patterns of work on patient outcomes. However, published data [4] including the National Confidential Enquiry into Patient Outcome and Death reports [5], [6] suggest that delays in review of patients and in obtaining senior opinion can contribute to avoidable deaths. This would suggest that medical patterns of work should be designed to improve outcomes. The published data also suggests that patients who are admitted to hospital either at weekends as an emergency or out of routine working hours have a higher mortality [7], [8], [9]. Previous small studies suggest that patterns of care delivery with greater Consultant involvement may be associated with better outcomes [10], [11]. This study explores the potential relationship between the recommended Consultant patterns of work and clinical outcomes for patients admitted as a medical emergency.

The aim of the study was to further investigate whether systems of Consultant cover that provide more continuous care were associated with better outcomes for adult patients admitted to hospital as an acute medical emergency. The study hypothesis was that better patient outcomes are associated with more continuous Consultant care as defined by the previously recommended service configurations for adult acute medical admissions [1].

## Methods

### Ethics Statement

The study involved no change in the management of patients and no access to identifiable patient level data. The study was submitted for ethical review and was approved by the Royal Free Hospital and Medical School, London Research Ethics Committee (Ref 09/H0806/64).

A nationwide survey of the Consultant patterns of work in acute hospitals was undertaken by the Royal College of Physicians London. This data was then linked with data derived from Hospital Episode Statistics (HES) for patients admitted to each participating hospital as an acute medical emergency. The patient outcomes analysed were overall adjusted case fatality rate, the ratio of observed to expected numbers of deaths of those admitted at weekends compared with weekdays, hospital length of stay and hospital readmission rates within 7 and 28 days for acute medical admissions.

### Survey

The survey of Consultant working patterns was carried out between February–April 2010. All acute hospitals in England were invited (via Acute Medical Consultants or Trust Medical Directors) to complete a previously piloted, web-based questionnaire (via ‘SurveyMonkey’ [12]) consisting of 65 questions. The questionnaire collected structured information about the organisation of senior medical cover for acute medical admissions. The responses to individual questions were then combined to form composite variables that best described optimal working arrangements related to more continuous Consultant cover, which were developed based on the initial pilot results [13].

### Analysis of Hospital Episode Statistics (HES)

Administrative hospital inpatient data (HES) from England was used for the analysis of the pre-defined hospital outcome measures. Data on adult (age ≥16 years) acute medical admissions (main specialty codes 300–499) for each participating hospital from the 12 month period from 1st April 2009 to 31st March 2010 were included. Mortality was defined as any patient coded as being dead on discharge. Re-admissions of the same patients were based on pseudonymous unique patient identifiers provided by the NHS Information Centre. The various outcome measures were aggregated to hospital level for analysis. The inpatient and mortality data were obtained through access arrangements agreed with Imperial College London. The following outcome measures were used for the analysis:

- Adjusted Case fatality rate (aCFR) for emergency medical admissions.
- The ratio of observed to expected numbers of deaths of those admitted at weekends compared with weekdays.
- 7 day emergency re-admission rates.
- 28 day emergency re-admission rates.
- Mean total length of stay.

### Case Mix Adjustment

Recognising the potential impact of case mix on the predefined outcomes the data were adjusted for:

- Disease severity and complexity using the Charlson comorbidity index [14]. This was calculated for each admission using the Deyo coding algorithm [15]. These were then used to calculate a weighted mean Charlson comorbidity index for each hospital.
- Age. Each outcome measure was adjusted for an age effect calculated using linear regression.
- The Index of Multiple Deprivation [16]. This was calculated as the average of the deprivation scores of the Lower Super Output Areas (LSOAs) [17] (small geographical areas with an average of 1500 people) of the residences of the patients admitted to each hospital.

A step-wise multivariate regression analysis was then applied to each of the adjusted outcome variables with the service variables as the dependant variables using SPSS (version 17).

We did go one step further in relation to the aCFR by including the rates of non-consultant doctors working in General medicine (using the NHS Workforce data from 2010 [18]) i.e. to assess any relationship between the aCFR and the numbers of admissions per each whole time equivalent House Officer, Senior House Officer, Foundation Year 2 trainee and Registrar.

## Results

105 hospitals completed the Consultant survey. The hospital site code field was not completed in the HES data set for 14 hospitals. This meant that in these cases the outcome measures were only available for the overall trust, which may encompass more than one acute hospital. Given the variation that exists between trust sites, it was agreed to exclude those 14 hospitals where there was insufficient site-specific HES data. This reduced the hospital sample size to 91 for the overall analysis. As shown in **Table 1**, the trusts included in the analysis were representative of all the Strategic Health Authorities (SHA) in England with participation ranging from 53% to 63% in each SHA region.

**Table 1:** Hospitals included in regression analysis by Strategic Health Authority (SHA).

	Total Trusts	Trusts with hospitals with complete Survey Response and HES data	
SHA	Number	Number	%
East Midlands SHA	9	5	56%
East of England SHA	17	9	53%
London SHA	28	16	57%
North East SHA	8	5	63%
North West SHA	24	14	58%
South Central SHA	9	5	56%
South East Coast SHA	13	8	62%
South West SHA	20	12	60%
West Midlands SHA	17	10	59%
Yorkshire and The Humber SHA	13	7	54%
**Total**	**158** *	**91**	**58%**

* The total number of trusts in table 1 is taken from HES data of all trusts (or equivalent) accepting emergency admissions.

### HES Data

Patient data was available for 1.3 million Adult Emergency Medical admissions in the participating hospitals in the study period. Baseline characteristics of the hospitals and demographics are shown in **Table 2**.

**Table 2:** Characteristics of hospitals and Acute Medical Emergency patient spells included in the study.

Hospital characteristic	Mean	Min	Max
**No. of beds**	606	230	1187
**No. of Adult Medical Emergency admissions per annum**	13,550	5,479	27,440
**Mean Age**	65	58	70
**Percentage of patients aged ≥75**	31%	14%	52%
**Female: Male ratio**	1.1: 1	0.8∶ 1	1.3∶ 1
**Mean Length of Stay (LOS) days (Spell)**	8.5	5.2	12.1

### Outcome Measures

The statistics describing the overall hospital outcome measures are shown in **Table 3**.

**Table 3:** Statistics of Hospital Outcome Measures relating to Acute Medical Emergency patient spells.

HOSPITAL OUTCOME MEASURES	MEAN	RANGE
**Overall adjusted Case Fatality Rate (aCFR)**	4.3%	2.5%–6.4%
**aCFR of those discharged in 0–1 days**	3.3%	1.2%–6.3%
**aCFR of those discharged in 2–3 days**	5.7%	1.7%–9.7%
**aCFR of those discharged in 4–6 days**	6.3%	1.7%–10.0%
**aCFR of those discharged in 7 or more days**	12.2%	7.9%–19.5%
**aCFR of weekend admissions**	4.8%	2.5%–7.7%
**aCFR of weekday admissions**	4.2%	2.1%–6.6%
**Ratio of aCFR of those admitted at weekends compared with weekdays**	1.15	0.89–1.49
**7 day readmission rate**	8.9%	5.6%–13.3%
**28 day readmission rate**	12.9%	9.8%–17.0%
**Mean LOS (days)**	8.5	5.20–12.10

Table 3 data demonstrates that an increased adjusted case fatality rate is associated with longer hospital length of stay (eg. Mean aCFR is 12.2% in those discharged in 7 or more days vs 3.3% in those discharged in 0–1 days); a higher aCFR also occurs among those admitted at the weekend (4.8%) compared with a weekday (4.2%) (P<0.01).

### Multiple Regression Analysis

The results of the multiple regression analysis on the main patient outcome variables are as follows. Effect sizes have not been included as we are only showing associations and the inclusion of such estimates may be misinterpreted as evidence of cause and effect relationship:

A higher Charlson comorbidity index (ie greater co-morbidity) was associated with:

- A longer hospital LOS (p<0.01)
- Higher aCFR for patients staying in hospital 4 days or more (p<0.01),
  Admitting Consultant continuous presence for a minimum of 4 hours per day was associated with:
- Reduced overall hospital aCFR (P<0.01). This was significant for 0–3 day LOS aCFR (P<0.01) and long stay (>7 days) aCFR (P<0.01).
- Lower 28 day readmission rate (P<0.01).

This pattern of working was adopted by 64 of 91 hospitals in this study.

Admitting Consultant continuous presence was defined in the questionnaire as one or more Consultants being immediately available during an on-call period, as opposed to only being available on request or for ward rounds. According to the recommendations, Consultants should be based within the admissions area or in close proximity. A continuous presence would be defined as equivalent to the proximity of an Intensive Care Consultant to the majority of Intensive Care Units during a day shift in the UK. An ‘admitting Consultant’ could be from any medical specialty, including Acute Medicine.

All-inclusive Consultant pattern of working was associated with:

- Reduced excess hospital aCFR of weekend versus weekday admissions (P<0.05).
  This all inclusive pattern was only adopted by 29 of the 91 hospitals in this study.
  ‘All-inclusive’ was defined as a combination of the key Acute Medicine Taskforce recommendations describing how an admitting medical Consultant should function when on call:
  - On call sessions should be protected. All other duties, such as outpatient clinics and procedures should be cancelled.
  - An admitting Consultant should be on call for two or more continuous days in a row.
  - There should be two or more ward rounds per day seeing all patients within the Acute Medical Unit.
  - These arrangements should be in place 7 days a week.
  In hospitals with higher numbers of acute medical admissions:
- Overall aCFR was reduced (p<0.03). This association with aCFR was significant for patients staying in hospital for 4 days or more (p<0.01), but not 0–3 days.
- The 7 day readmission rate was lower (p<0.02).
- The mean length of stay (LOS) was higher (p<0.02).

The average number of acute medical admissions was 39. Thus, hospitals with 40 or more acute medical admissions daily were classified as having a higher number of admissions.

Hospitals with higher numbers of hospital admissions were shown to have higher numbers of Consultants contributing to the care of acute admissions. However, hospitals with higher numbers of admissions were not more likely to adopt patterns of work consistent with a prolonged continuous Consultant presence or all-inclusive consultant pattern of working.

The mean length of stay was not related to any Consultant staffing variables in this study.

The analysis of other medical staff by grade (House Officer (including Foundation trainees), Senior House Officer and Registrar) showed no associations with aCFR or other outcome variables.

## Discussion

To our knowledge, this is the first paper that explores a potential relationship between recommended Consultant working patterns [1] and patient outcomes in acute medical care across a range of health care providers. Multiple regression analysis provides evidence only of associations, and any potential causal relationship would strictly need to be confirmed by interventional trials.

The results show that having a continuous admitting Consultant presence on the acute medical unit is associated with reduced adjusted case fatality rates in hospital and the association is most apparent with early death (<3 days) in hospital. This outcome supports the published recommendations about early Consultant review in patients admitted as an acute medical emergency as outlined in the Acute Medical Task Force report and is consistent with the findings in the recent National Confidential Enquiry into Patient Outcome and Death (NCEPOD) reports [1], [5], [6] **.** Our additional analyses of the impact of other junior medical staff did not demonstrate associations with lower aCFRs with the other grades of doctors per 100 admissions. This appears to emphasise the role of the consultant as a senior decision maker in the acute medical setting. A prolonged, continuous Consultant presence is also associated with reduced 28 day re-admission rates, which is an important outcome if we are to improve patient experience and reduce the demand on acute inpatient hospital beds.

The ‘All-inclusive’ Consultant pattern of working reflects the attempts to improve continuity of care for patients admitted as acute medical emergencies. The results suggest that hospitals adopting this pattern of more continuous working and more regular review of patients experienced lower excess weekend mortality rates. However it is notable that at the time of the survey only one third of the hospitals had adopted this recommended pattern of work.

The hospitals studied appear to be a representative sample of acute NHS hospitals in England as they were distributed relatively uniformly across all SHAs and within each SHA over 50% of acute hospitals contributed data. In addition, the overall data is consistent with published data [19] in terms of age, male: female ratios, the number of medical admissions, readmission rates and hospital length of stay as defined by hospital spell of care. The HES data for the studied hospitals shows that aCFR increases with longer patient stays in hospital admissions and confirms the excess mortality for patients admitted at weekends [7], [10], [20]. Our data demonstrates a similar effect size on weekend mortality compared to Aylin’s study [21].

Interestingly in this multivariate analysis, those hospitals with higher numbers (40 or more per day) of acute medical admissions were associated with a reduced aCFR. This is consistent with previous studies which have indicated that larger hospitals have improved outcomes [22], [23]. Hospitals with higher numbers of admissions were also associated with a reduced number of 7 day readmissions but this was not reflected in the 28 day readmission rate routinely measured in NHS hospitals. Hospitals with larger numbers of admissions also had a longer mean LOS. As intuitively expected, hospital with higher number of acute medical admissions had more Consultants caring for acute admissions, but they were not more likely to adopt recommended Consultant working patterns. These associations with higher numbers of medical admissions, independent of Consultant working patterns, suggest a need for more study in this area.

One purpose of this study was to demonstrate that linking HES data with contemporaneous staff survey data at a hospital level can provide insights into patterns of care and highlight areas for more in depth study. This study has demonstrated a novel way of both obtaining “soft” information from a representative sample of English NHS acute hospitals to combine with “hard” HES data to assess associations of staffing with hospital outcomes. This approach may be applicable to other aspects of hospital care.

The limitations of this study are that it was retrospective and compares two different data sets. Apart from the investigation into aCFRs and other medical staff, it does not take into account other staffing levels including nursing, allied health professionals and pharmacists. Other potential confounding factors such as access to diagnostic services were also not analysed. However, reasons to accept the associations we found as reliable include the facts that: this study includes data from 91 hospitals in England and includes over 1.3 million patient spells of care; the demographics of the patients are consistent with previously published data [8] and the participating hospitals are geographically distributed throughout England. In addition, the overall HES data confirms the recognised increased aCFR in those admitted at weekends versus weekdays [7], [8].

It is possible that the described models of Consultant care reflect other aspects of the organisational systems and processes and this merits further investigation. However, the associations are in line with the previous recommendations of the Royal College of Physicians London for Consultant patterns of working while undertaking acute medical take. Our findings are also consistent with the NCEPOD reports [5], [6] recommendations regarding the need to involve senior clinical decision makers promptly in the review and management of the acutely unwell patient. The survey data shows that many hospitals have still not adopted these recommendations. The associations in this study would add a further layer of evidence that NHS hospitals admitting acute medical emergencies should develop plans to implement these recommendations to improve patient outcomes. This will require support from Commissioners. Of note, in 2012 NHS London developed commissioning standards that support the recommended model of care [24]. To improve clinical quality we must continue to monitor outcomes for patients admitted to hospital as an emergency at a weekend and out of hours and use this to support continuous service improvement.

Given the complexity of emergency medical care we would recommend further in-depth studies in this area to more fully understand the role of Consultants and the essential role of other health care professionals, as well as access to diagnostic and interventional services, to help establish the best possible models for acute medical care.

### Conclusions

This study demonstrates an association between recommended working patterns for medical Consultants on call and better outcomes in patients admitted as medical emergencies. These results are in line with published recommendations for Consultant working patterns and can be summarised as a drive to improve continuity of care with early senior decision input. Individual hospitals and their parent Trusts should be working towards systems encompassing these recommendations, in order to deliver improved outcomes for patients.

The results, along with those of the earlier RCP reports have also generated a number of intriguing hypotheses which should prompt further research.
